# A nationwide program to improve clinical care quality in the Kyrgyz Republic

**DOI:** 10.7189/jogh.10.020418

**Published:** 2020-12

**Authors:** John W Peabody, Klara Oskombaeva, Memerian Shimarova, Venera Adylbaeva, Kanzaada Dzhorupbekova, Irina Sverdlova, Venera Shukurova, Zhyldyz Abdubalieva, Natalya Gagloeva, Ainura Kudayarova, Aizhamal Asanbekova Mukanbetovna, Nurgul Shoonaeva Dzhumagazievna, Violetta Vibornykh, Mimoza Satybaldieva Zhorobekovna, Enrico de Belen, David Paculdo, Diana Tamondong-Lachica, Daniel Novinson, Czarlota Valdenor, Gyorgy Fritsche

**Affiliations:** 1 QURE Healthcare, San Francisco, California, USA; 2 University of California, San Francisco, California, USA; 3 University of California, Los Angeles, California, USA; 4 World Bank, Washington, D.C., USA; 5 Ministry of Health, Bishkek, Kyrgyz Republic; 6 Kyrgyz State Medical Institute of Retraining and Further Training named after S. B. Daniyarov, Bishkek, Kyrgyz Republic; 7 National Center of Cardiology and Therapy named after Academician M. M. Mirrahimov, Bishkek, Kyrgyz Republic

## Abstract

**Background:**

To assess baseline quality of care in the Kyrgyz Republic in 2019 and determine the effect of online simulated patients in changing doctors’ practice in three specific disease areas: non-communicable disease, neonatal/child health, and maternal health.

**Methods:**

Over 2000 family health, pediatric, neonatology, therapy, and obstetric-gynecologic doctors from every rayon (district) hospital and at least one associated family health (Primary) care clinic participated. To adequately scale the project, the Ministry of Health used online simulated Clinical Performance and Value (CPV) vignettes. All doctors cared for the same set of patients in their clinical area. Over eight months in 2019, we gathered three rounds of CPV data in seven oblasts.

**Results:**

Overall quality scores were highly variable at baseline (59.2% + 13.5%). After three rounds the average score increased 6.5% (*P* < 0.001). By the end of round three, the lowest scoring oblast was providing higher quality care compared to the highest scoring oblast in the initial round (64.2% in round 3 vs 62.4% in round 1), indicating greater adherence to the evidence base. Additionally, family health doctors ordered 26% fewer unnecessary tests (*P* < 0.05), while specialists ordered 39% fewer unnecessary tests (*P* < 0.05). If trends continue, this translates into a net annual savings of 63 million Kyrgyz som.

**Conclusions:**

This study demonstrates serial measurement of care provided by over 2000 physicians in the Kyrgyz Republic can be improved as measured by CPVs. This project may be a useful template to improve health care quality at a national level in other low- and middle-income country settings.

A major challenge for any large health care system – whether local, regional, national, or multi-country – is the ability to measure clinical practice of all their providers affordably and at-scale. Subsequent, and fully related, is how to improve quality and monitor this across the system over time to reduce the inevitable variability in care [1]. These problems are notoriously complex even in developed nations and especially so in low- and middle-income countries (LMICs) [2]. Previous studies have shown that performing a cross-national sampling of baseline provider care is possible [3].

The first step in addressing this challenge is conceptual: deciding upon a meaningful and locally relevant framework by which to measure health care quality. The well-established Donabedian framework [4,5] to improve the quality of care, accounts for other inputs on health care quality, links health care *access* and *structural*-level resources, which in turn creates a foundation to deliver care by providers, referred to as the *process*-level of quality, which in turn is linked to *outcomes* [4].

The next challenge is executional – how to implement national-level measurement and improve care quality at scale, especially in LMICs where remote regions face issues of accessibility, differential skills or resources. One proven approach to measuring health care quality at scale is using simulated patient cases to evaluate the clinical behavior of health care providers. Clinical Performance and Value vignettes (CPVs) have been successfully employed to measure health care quality in developing-world settings, including the Philippines, China and other parts of Asia and throughout Eastern Europe [2,6].

In this study from the Kyrgyz Republic, we used the Donabedian framework and CPVs to evaluate all public-sector physicians across Rayon district hospitals and most of the referring family medicine clinics. Overarching goals of the project included improving clinical quality, reducing unnecessary clinical variation, and reducing health care spending. To achieve these goals, project team members worked to implement a project with five key attributes: feasibility, affordability, timeliness, responsiveness, and sustainability.

We report on three rounds of CPV data collected from approximately 2000 respondents completed serially in all oblasts in Kyrgyzstan. The cases focused on three clinical areas: (1) neonatal and child health, (2) obstetrics, and (3) noncommunicable cardiovascular diseases. In each clinical area, we randomly assigned eight clinical cases complete with individual, real-time feedback and end-of-case feedback based on evidence-based guidelines. The individual feedback provided training of the physicians and tracking of physician practice change. The end-of-case feedback provided comparative, motivational metrics among oblasts and the rayon facilities to track progress at the aggregate level.

## METHODS

### Setting

The *QURE-Quality Improvement in Clinical Care for Kyrgyztan *(QuICCK) *Project*, under the aegis of the World Bank and the government of the Kyrgyz Republic, is a partnership between QURE Healthcare and the Ministry of Health of the Kyrgyz Republic (MoH) to improve quality of health care delivered in rayon hospitals and attached family medicine centers (FMCs) and general practice centers (GPCs). The QuICCK project began in April 2019. The three rounds of data collection included in this study started in June 2019 and ended five and a half months later in November 2019.

### Epidemiology and disease selection

The study focused on three clinical areas designated by the MoH based on the burden of disease in the Kyrgyz Republic: 1) Neonatal and Child Health (NCH), 2) Maternal Health (OB), and 3) Non-Communicable Cardiovascular Diseases (NCD). The three clinical areas encompassed both ambulatory/outpatient and inpatient settings. (See **Box 1** for details on disease selection.)

Box 1 **Epidemiology and disease selection.**

An estimated 6.3 million children and young adolescents died in 2017 worldwide, mostly from preventable causes, with 5.4 million of these deaths accounted by children under 5 years of age. Under five mortality rate (U5MR) is high in central Asia. Specifically, the U5MR has remained more than 4 times higher in the Kyrgyz Republic compared to other countries in Europe. Moreover, the infant mortality and neonatal mortality contribute to 90% of the U5MR in the country [7].

The maternal mortality rate (MMR) is too high in the Kyrgyz Republic at 60 per 100 000 live births – 10 times higher than that in western Europe [8]. The main causes of maternal mortality in this region are direct causes resulting from hemorrhage, followed by hypertension, and sepsis [9,10].

Non-communicable diseases were responsible for 86% of all deaths in the Kyrgyz Republic. Cardiovascular diseases accounted for 54% of the overall mortality, of which ischemic heart disease and cerebrovascular diseases contributed one-half and one-third, respectively [11].

### Facilities

The foci of this study were all the government territorial district (Rayon) hospitals and the primary care facilities in the standalone, outpatient FMCs or GPCs which are outpatient clinics co-located with a rayon hospital. Members of the RBF secretariat (the project implementation unit), compiled the list of all the facilities in the country, including rosters of doctors. A total of 120 institutions were included: 40 territorial hospitals, 51 FMCs, and 29 GPCs.

### Providers

All physicians who provided NCD, pediatric, neonatal, or obstetric care from each of the participating health facilities were enrolled in this study. The physicians were subsequently classified by service line: therapists (internal medicine/general practice), pediatricians, neonatologists, obstetricians, and family health (FH) doctors. Medical directors and deputy directors of all facilities were also included to further promote engagement.

### Measurement of quality

**Figure 1:**
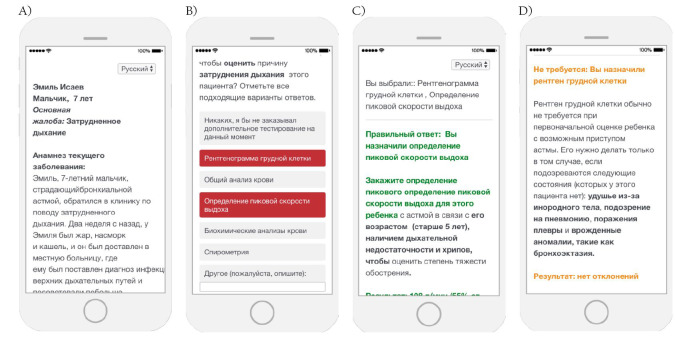
Clinical Performance and Value (CPV) sample screenshots. **Panel A**. Introduction screen. **Panel B**. Workup screen. **Panel C**. Feedback screens.

The QuICCK project collected data on quality of care using a facility-level and physician-level survey for the structural measures and the Clinical Performance and Value (CPV^®^) vignettes for the clinical practice and care process measures (**Figure 1**). QuICCK investigators developed 24 CPV vignettes, eight in each identified clinical area, following WHO guidelines and local context. Case development included provision of real-time doctor-specific feedback in areas where the participant either did not provide evidence-based orders or provided orders that were not supported by the evidence base. As individual doctors completed each case, the software delivered specific relevant portions of feedback based on that doctor’s choices. In addition, feedback on the aggregated results was given immediately prior to starting the next round of cases. See **Box 2** for greater methodologic details. Cases were loaded on the Qualtrics® (www.qualtrics.com) platform’s Survey Tool, which functions on both desktop and mobile phones, and importantly eases access in remote sites for doctors in LMICs who may not have Internet access or a computer.

Box 2 **Methodological survey and measurement details of the QuICCK Project.**

*Structural measures*. Two separate surveys were conducted to gather data on structural attributes that may affect the outcomes of care: a facility survey and an individual provider survey. These surveys were done to determine the facility and physician characteristics that influenced the CPV measured quality of care.

The facility survey was conducted through in-person interviews in each hospital and primary health care facility by the field teams. Data collected through this survey included information on personnel and management, health information systems, services provided, patient mix, material resources, and financial resources. The individual provider survey was self-administered by each participant through the same online platform used to administer the CPVs and collected information on demographics, amount of training, patient load, as well as personal insights into the Kyrgyz health care system. Understanding these factors helps provide a more complete picture of the quality of health care delivery in the Kyrgyz Republic. These surveys were done once for each facility and provider.

*Quality of care process measures*. The measurement of care quality in this study used CPV vignettes – simulated patient cases – which were designed and developed by QURE Healthcare and reviewed with local clinical experts designated by the MoH. The simulated CPV patients are online clinical cases that recreate a typical patient encounter that were originally validated against actual practice [12,13]. Their utility has been validated both against actual changes in practice and in patient outcomes over time and across multiple settings [14-18].

The investigators developed 24 CPV vignettes: 8 cases for each of the 3 identified clinical areas (NCD, NCH, and OB). For NCH care, there were 2 cases each for neonatal jaundice, neonatal sepsis/pneumonia, respiratory failure from bronchial asthma, and diarrhea with dehydration. For OB, there were 2 cases each for septic shock from complicated urinary tract infection, post-partum hemorrhage, preeclampsia, and puerperal sepsis. For NCD, there were 2 cases each for type 2 diabetes mellitus, ischemic stroke, acute myocardial infarction, and chronic obstructive pulmonary disease.

To be able to compare family health (FH) doctors to specialists, each patient presented to the outpatient/ambulatory level. To evaluate specialty care, each outpatient required care in the hospital and thus needed to be referred for admission and inpatient management where only specialists care for patients in Kyrgyzstan. Every case starts by presenting the providers with a patient history (ie, a chief complaint, present history, past medical history, family and social history) and the pertinent physical examination findings for an undisclosed clinical condition. All providers begin their cases with three interactive domains to determine the patient’s condition and formulate the necessary management by: (1) ordering diagnostic workup, (2) generating a diagnosis, and (3) providing an initial, disease-specific treatment plan (including disposition of the patient either to home, in-clinic care, or hospital). At this point, care for the patient diverges. The specialist doctors are asked to care for the patient in the hospital and make additional management decisions and after the patient improves and is discharged from the hospital, the specialist is asked to provide outpatient follow-up and preventive care. The FH doctors, who do not provide hospital-based care, are given a summary of the patient’s hospital course, and then also asked to provide outpatient follow-up and preventive care.

Primary care doctors took care of all three cases. Specialists only cared for cases in the respective clinical areas: pediatricians and neonatologists were limited to pediatric or neonatal cases, obstetricians were limited to obstetric cases, while therapy doctors (including facility directors and deputy directors) were limited to taking NCD cases. All cases were randomly assigned prior to the beginning of round 1.

Each round of the QuICCK study occurred about two months apart and, after the cases were completed, had two feedback components: (1) real-time, immediate, individual feedback during CPV administration given to the providers on the care of their cases, and (2) feedback on individual and facility scores from the previous round including highlights of common areas of poor quality/high variation for each individual case type.

Each round of data collection takes about 4 weeks. Round 1 began on 17 Jun 2019 and ended in 05 August 2019, with 1941 providers reached. Round 2 began on 26 August 2019 and ended in 24 September 2019 with 1994 providers participating. Round 3 data collection ran from 21 October 2019 to 02 November 2019 and involved 2136 providers. Subsequent rounds will be ongoing and follow the same schedule. In total, 2347 unique providers have already participated in QuICCK.

### Data analysis

Providers were required to indicate how they would usually manage each case, just as they would do in a real-life scenario. Caring for each simulated patient takes about 20 to 30 minutes to complete. Completed CPVs were scored against pre-determined, evidence-based quality criteria as specified by national Kyrgyz and WHO guidelines for each clinical condition. Items with greater importance (such as primary diagnosis and definitive treatment) were given the most weight in scoring. In cases where local and WHO protocols were not existent, guidelines from American and European medical societies were utilized. The CPVs were also adapted to the local clinical setting and health care practices in the country. Individual domain scores for workup, diagnosis, and treatment ranged from 0% to 100%, and an overall score was also calculated based on how the provider performed across all domains, with higher percentage scores reflecting greater alignment with evidence-based practice recommendations. Although it would be tempting to compare scores between different case types, we do not do so because cases are inherently different obviating our ability to compare cases properly.

Since we gathered census data, there were no adjustments necessary for sampling. We used descriptive statistics and *t*-tests to identify significant differences in CPV scores between facilities, regions, and rounds. For the structural measures of quality, we performed descriptive statistics using χ^2^ analyses for binary outcomes and Student’s *t* test for continuous outcomes. For multivariate modeling, we performed linear regression analyses. All statistical analyses used STATA v14.2 (StataCorp, College Station, Texas, USA).

## RESULTS

### Provider characteristics

Over the three rounds of QuICCK, 2347 doctors from seven oblasts completed the surveys and took CPVs in at least one round of the study. Overall, 23.4% of the doctors identified as male, and the average age of all doctors was 47.6 ± 14.3 years (**Table 1**). Overwhelmingly, most (90.5%) received their training in Kyrgyzstan, with much of the rest taking their medical education in another part of the former USSR (5.4%). By doctor type, 51.4% practice as FH providers, while the rest were a mix of specialists including obstetricians/gynecologists (11.2%), pediatricians/neonatologists (11.1%), therapists (18.2%), and deputy directors (8.0%). Specialists compared to FH doctors tended to be younger (44.7 ± 14.5 years for specialists vs 50.4 ± 13.4 years for FH doctors) and almost twice as likely to be male (30.3% for specialists vs 16.8% for FH) except for obstetricians/gynecologists where only 7.6% are male, compared to 44.2% of therapists. Unsurprisingly, specialists tended to be less represented in more rural oblasts. For example, in Naryn and Talas oblasts, specialist providers represented only 37.0% and 34.6% of the physician population tested, respectively, as compared to the larger oblasts of Osh and Jalal-Abad, where specialists made up more than 50% of the population (58.4% and 51.2%, respectively).

**Table 1:** Provider characteristics

	All		Family health		Specialists		
	**Value**	**SD**	**Value**	**SD**	**Value**	**SD**	***P*-value**
N	2,347		1,206		1,141		–
Male, %	23.4%	–	16.8%	–	30.3%	–	<0.001
Age	47.6	14.3	50.4	13.4	44.7	14.5	<0.001
**Doctor type:**							
Family health	51.4%	–	100.0%	–	–	–	–
Obstetrician/gynecologist	11.2%	–	–	–	23.1%	–	
Pediatrician/neonatologist	11.1%	–	–	–	22.9%	–	
Therapist (internal medicine)	18.2%	–	–	–	37.5%	–	
Deputy director	8.1%	–	–	–	16.6%	–	
Years of medical education	8.9	9.1	9.6	10.1	8.1	7.8	<0.001
Years of practical training	4.5	8.8	4.6	9.1	4.4	8.5	0.472
Hours worked per week	47.1	20.4	45.9	17.4	48.4	23.1	0.004
**Country of medical education:**							
Kyrgyz Republic	90.5%	–	88.5%	–	92.6%	–	0.008
Other part of former USSR	5.4%	–	6.4%	–	4.4%	–	
Other	4.1%	–	5.2%	–	3.1%	–	
**Oblast:**							
Batken	12.6%	–	12.0%	–	13.2%	–	<0.001
Chui	18.3%	–	21.3%	–	15.2%	–	
Issyk-Kul	8.7%	–	9.8%	–	7.5%	–	
Jalal-Abad	20.9%	–	20.4%	–	21.4%	–	
Naryn	5.4%	–	6.7%	–	3.9%	–	
Osh	30.1%	–	25.1%	–	35.3%	–	
Talas	3.5%	–	4.5%	–	2.4%	–	
Unknown	0.6%	–	0.2%	–	1.1%	–	

SD – standard deviation

### Baseline CPV scores

At baseline (the first round), providers overall averaged 59.2% (SD 13.5%). Scores ranged from 6.3% to 100% with variation, as measured by the interquartile range (IQR), ranging between 50.0% to 68.0%. Examining the three different disease types, we see that providers scored 58.3% in the NCD cases, 57.3% in the NCH cases, and 62.1% in the OB cases.

Scores from the initial encounter, which all providers were required to perform – workup, diagnosis, and initial treatment – we found that FH doctors and specialists scored similarly (64.0% ± 20.4% vs 64.2% ± 20.6%, *P* = 0.802). By subdomain, we found FM doctors provided better evidence-based workup than their specialist doctor counterparts (workup: 51.2% for specialists and 47.0% for FM doctors, *P* = 0.007), but specialists provided better initial diagnosis and treatment (diagnosis: 69.5% for specialists and 62.4% for FM doctors, *P* < 0.001; treatment: 81.7% for specialists and 77.5% for FM doctors, *P* = 0.001)

The wide variation in care at baseline was seen regardless of specialty: the therapists scored 63.8% ± 19.8%, neonatologists/pediatricians scored 59.5% ± 24.4%, and OB-GYNs scored 69.4% ± 16.3%. For FH doctors, who care for all three patient types up to the point of being admitted, in the NCD and NCH case types, their scores were not significantly higher than their specialist counterparts (NCD cases: 65.2% vs 63.8%, *P* = 0.250; NCH cases: 60.5% vs 59.5%, *P* = 0.563). In the obstetric cases, however, FH doctors scored significantly lower than specialists (66.1% vs 69.4%, *P* = 0.006). A lot of this difference was due to FH doctors making the primary diagnosis at a significantly lower rate (53.7% vs 64.1%, *P* = 0.003).

Scores varied by region even in this relatively small country. By oblast, at baseline, the highest scoring was Naryn, where physicians scored on average 62.4% ± 12.4% across all cases, while the lowest scoring oblast was Osh, where scores averaged 56.9% ± 13.4%, a significant difference (*P* < 0.001) (**Table 1**). Among FH doctors, we still see the difference between oblasts with Naryn scoring highest (63.1% ± 12.5%) and Osh scoring lowest (59.7% ± 12.4%) (*P* < 0.001). Scores for the specialists followed a similar story.

In multi-variable regression analysis, after adjusting for oblast and doctor type (FH vs specialist), being female was the only significant physician characteristic associated with a higher score (+3.3%, *P* < 0.001) compared to their male counterparts – a finding we have seen in many other studies across the economic development spectrum [3,19,20]. Age showed almost no influence with an increase in CPV scores of 0.1% per decade increase in age. Facility characteristics had no influence, statistically, in determining the baseline CPV scores (*P* > 0.05) for all characteristics examined.

### Trends in CPV scores

**Figure 2:**
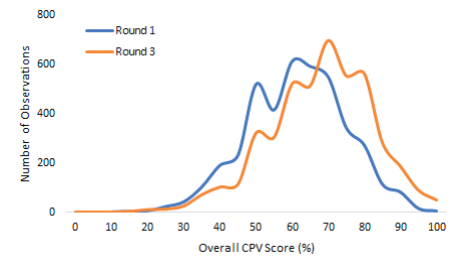
Comparison of overall Clinical Performance and Value (CPV) scores, Round 1 to Round 3.

Over time, with serial measurement and feedback, the doctors’ scores improved from 59.2% to 65.7% (on average) and the SD decreased from 13.5% to 12.5%. These are both statistically (*P* < 0.001) and clinically significant [14]. The IQR measure of variation decreased from a round 1 range of 50.0%-68.0% to a round 3 range of 57.1%-75.9%, indicating that the improvement was across all doctors (*P* = 0.042) (**Figure 2**). By case type, doctors scored 63.7% in NCD cases, 63.9% in NCH cases, and 69.6% in OB cases (*P* < 0.001 for all compared to baseline). In terms of variation reduction, there was a dramatic and significant reduction in obstetrics, where standard deviation decreased from 13.0% to 12.3% (*P* = 0.041) but no statistically significant reductions in the other case types after three rounds.

Again, looking at oblasts, the highest scoring oblast after three rounds shifted from Naryn to Talas which averaged 69.3%, compared to the lowest scoring oblast, Osh at 64.2% (*P* < 0.001) (**Table 2**). Importantly, the lowest scoring oblast in round 3 scored higher than the highest scoring oblast in round 1, demonstrating a shift in the overall quality of care and a greater national adherence to evidence base practice. Just as significantly by round 3, the variation in average oblast scores decreased from 5.6% (62.4% - 56.9%) to 5.1% (69.3% - 64.2%), a relative improvement of 9%. In regression analysis, at baseline and after the third round, we found no statistically significant difference in scores between oblasts (*P* = 0.120 and *P* = 0.558).

**Table 2:** Overall CPV Results by oblast, Round 1 to Round 3

	Round 1		Round 3			
**Oblast**	**Average score**	**SD**	**Average score**	**SD**	**Round 1 to Round 3 improvement**	***P*-value**
Batken	57.5%	14.3%	64.7%	14.7%	+7.2%	<0.001
Chui	60.4%	13.3%	66.5%	13.6%	+6.1%	<0.001
Issyk-Kul	61.1%	12.2%	68.3%	13.0%	+7.2%	<0.001
Naryn	62.4%	12.4%	66.3%	13.7%	+3.9%	0.002
Osh	56.9%	13.4%	64.2%	14.3%	+7.3%	<0.001
Talas	62.0%	12.9%	69.3%	12.8%	+7.4%	<0.001
All	59.2%	13.5%	65.8%	14.1%	+6.7%	<0.001

CPV – Clinical Performance and Value, SD – standard deviation

Confining our analysis to the initial evaluation, which was identical for both FH and specialist doctors, both groups improved from baseline to round 3 by almost identical amounts (+2.7% for FH doctors and +2.5% for specialists). By case type for the full specialist cases, we saw an increase in scores of +2.8% for therapists, +0.5% for neonatologists/pediatricians, and +4.4% for ob-gyns.

We repeated the multivariate analysis in round 3, which accounted for physician and facility characteristics, and again female doctors clinically outperformed male doctors by 3.0%, (*P* < 0.001). In addition, older physicians improved their scores more rapidly than younger physicians (+0.7% per decade of age, *P* < 0.001). The other facility characteristics remained nonsignificant.

### Issues of clinical interest

The improved diagnosis across all cases had the knock-on effect of improving the proper disposition of a patient who needed to be admitted to the hospital. At baseline, doctors properly dispositioned their patients 73.9% of the time, but by the end of the third round, this had improved to 89.0% (*P* < 0.001). In particular, the rates of doctors sending a patient home inappropriately, when they should have been admitted to the hospital, decreased by 36.2% (*P* = 0.026) and the NCH cases were sent home 48.3% less often (*P* = 0.008) (see **Table 3** and **Box 3** for areas of additional clinical interest).

**Table 3:** Issues of clinical interest

	Round 1	Round 3	*P*-value
**Primary diagnosis**			
Overall	63.9%	70.0%	<0.001
Non-communicable disease	85.7%	87.9%	0.075
Neonatal/child health	45.3%	61.8%	<0.001
Maternal health	55.7%	58.3%	0.170
**Correct disposition of patient after the initial encounter**	73.9%	89.0%	<0.001
**Non-communicable disease**			
Initial arterial blood gas of COPD patients	20.1%	38.9%	<0.001
Initial aspirin treatment of patients with STEMI	78.7%	89.4%	<0.001
Initial treatment of insulin for patients with hyperglycemia	37.5%	46.7%	0.008
Provide follow-up information to stop smoking	76.9%	88.4%	<0.001
**Neonatal/child health:**			
Initial bilirubin levels for neonatal patients presenting with jaundice	85.5%	94.1%	<0.001
Intravenous fluid with dextrose and half-normal saline in neonatal patients with sepsis	29.8%	50.4%	<0.001
Initiate short-acting beta agonist for asthmatic children with exacerbation	78.6%	84.6%	0.042
Offer oral rehydration for patients with acute diarrhea	67.3%	70.1%	0.443
**Maternal health:**			
Position pregnant mother with obstetric complications lying down	35.5%	56.2%	<0.001
Insert Foley catheter and monitor urine output	39.7%	55.7%	<0.001
Initiate magnesium sulfate for pregnant women with pre-eclampsia	87.9%	91.9%	0.084

COPD – chronic obstructive pulmonary disease, STEMI – ST-elevation myocardial infarction

Box 3 **Additional issues of clinical interest.**

Importantly, doctors showed improvements in focused clinical areas for each case type. For example, in patients with known COPD, doctors in round 3 appropriately ordered an initial arterial blood gas at nearly double the rate of round 1 (20.1% in round 1 vs 38.9% in round 3, *P* < 0.001) and they advised their patients to stop smoking more often in round 3 (76.9% vs 88.4%, *P* < 0.001). For the neonatal/child cases, doctors ordered appropriate intravenous hydration for neonatal patients (from 29.8% up to 50.4% of the time, *P* < 0.001) and were more likely to order oral rehydration for pediatric cases with acute diarrhea and dehydration (67.3% vs 70.1%, *P* = 0.443). In obstetrics, for a woman of almost 36 weeks gestational age with pre-eclampsia, the diagnosis improved from 32.9% to 41.6% (*P* = 0.102) and the use of oxytocin to induce labor expanded from 50.0% to 71.4% (*P* = 0.107).

At baseline, doctors made the correct primary diagnosis 63.9% of the time; this improved to 70.0% by round 3, an improvement that is both statistically and clinically significant (*P* < 0.001) (**Table 3**). Notwithstanding, we also found that there is still a great deal of room for improvement. In the obstetric cases, despite this improvement, doctors were still only able to make the correct diagnosis just over half the time (55.7% in round 1 and 58.3% in round 3; *P* = 0.170). By contrast, diagnostic accuracy in the NCD cases, which started with a high correct diagnosis rate at baseline improved, albeit slightly, by the third round (85.7% to 87.9%; *P* = 0.075). The greatest improvements were in the NCH cases, which started at a correct diagnosis rate of less than half, improved significantly (45.3% to 61.8% in round 3 (*P* < 0.001).

### Cost benefits of higher quality

We measured the amount of unnecessary workup (testing and imaging studies) ordered by the doctors. FM doctors and specialists together ordered an overall average of 1.4 unnecessary tests per case at baseline. For FM doctors, who ordered on average 1.3 unnecessary tests in Round 1, by round 3, they were ordering 26% fewer unnecessary tests (*P* < 0.05). For specialty doctors, therapists ordered 1.7 unnecessary tests, obstetricians/gynecologists ordered 0.9, and pediatricians/neonatologists ordered 1.3 in Round 1. By round 3, unnecessary diagnostic test ordering had decreased by 22% for therapists, 50% for obstetricians/gynecologists, and 45% for neonatologists/pediatricians (*P* < 0.05 for all).

Across all cases, the average number of necessary diagnostic workup items was 0.7 (range 0 to 5). The 0.7 needed vs 1.4 unneeded items means approximately two-thirds of all tests ordered were unnecessary. Per the WHO, the average annual out-of-pocket expenditure for a diagnostic test was 176 Kyrgyz som (US$ 2.28) per person in 2014 [11]. As the current population of Kyrgyzstan is approximately 6.4 million people, approximately 1.126 billion Kyrgyz som (US$14.57 million) were spent in out-of-pocket diagnostic tests. Assuming that two-thirds of all real-world tests were also ordered unnecessarily in Kyrgyzstan, 751 million som (US$ 9.72 million) were wasted on these tests. Approximately 2000 doctors, or one-sixth of all doctors in Kyrgyzstan, have participated in this project. By extension, 126 million soms (US$ 1.63 million) of the unnecessary diagnostic tests are attributable to the QuICCK participating doctors. Applying an ultimate 50% decrease in unnecessary testing, this equates to a direct savings of 63 million som (US$ 815 000) per year for the ~ 2000 doctors who have participated in the QuICCK project.

## DISCUSSION

We have previously argued that improving quality is the fastest and most effective way to improve the health status of individuals and populations [21]. Access and structural measures are important but, in the widely accepted structure→process→outcomes framework, far removed from the patient and the clinical care they receive. The problem, for years has been a scalable, repeatable accurate measure of clinical practice [22].

This study reports on the nation-wide roll out of a validated method, CPVs, used to measure the quality of clinical practice at national scale in the Kyrgyz Republic. The goals of this project, laid out at the beginning, were to improve clinical quality, reduce unnecessary clinical variation and reduce health care spending. To achieve these goals, project team members worked to implement a project with five key attributes: demonstrable feasibility, timeliness, relevance, responsiveness to feedback, and local sustainability.

Over the course of only 7 months, all of the Kyrgyz doctors working in the rayon (district) hospitals and the referring primary care clinics completed simulated cases, received their feedback and were benchmarked against their peers. This process was done three times, showing the feasibility and scalability of serial measurement with feedback.

The baseline findings were, unsurprisingly worrisome: there was wide variation in practice regardless of specialty. For basic care, which we codified by having specialists and FM doctors do the same initial assessment, there was little difference in the average quality of care. The most worrisome (relevant) finding was how much variation there was in care services. Simply put, if a patient went to one of the doctors in this study who did not know what they were doing, they would not be diagnosed or treated correctly. They would not be admitted to the hospital nor they would not be given lifesaving intravenous therapy, oxygen, or have labor induced. They were twice as likely to order an expensive, unnecessary test ordered as not to order a needed test leading to more misdiagnosis and ineffective treatment at an extraordinary cost to the individual and to the country. On a brighter note, there are already a cadre of clinicians throughout Kyrgyzstan practicing high quality medicine, servicing as examples of what evidence-based care looks like.

Happily, scores improved steadily and consistently in just a few short months, from baseline to round 3, achieving statistical significance in overall quality, in both types of providers, for different diagnoses and many therapeutic areas. More importantly, the variation also started to decline. With ongoing rounds occurring every two to three months even higher quality and lower variation is a reasonable expectation, something we have seen in other large-scale projects in the Philippines and in the United States [15,21]. In Kyrgyzstan we describe this as the best oblast (region) in round 1 did not do as well as the worst region in Round 3.

Introducing CPVs and getting full participation is another exciting local accomplishment, but this only tells half the sustainability story. After only three rounds, the technology and, more importantly, the capability to use the technology has been transferred to the government and the Kyrgyz State Medical Institute of Retraining and Further Training (MIR&FT). A dedicated team, without specific financial emoluments at the MIR&FT, has taken on the task and implemented round 4 (round 5 and 6 is planned) at the time of this writing. With only case 8 cases per disease area, it is obvious to the MIR&FT that we will run out of cases soon. This task too, of developing and writing the cases, has been taken on by the MIR&FT leadership and clinical experts. The other element of sustainability, often unnoticed, is performing the data analytics. The software program and the analytic framework have been transferred so that feedback reports, benchmarking and trend data can be produced by a dedicated post graduate team.

There are shortcomings to be sure. Perhaps the most worrisome is the point of failures that will occur if there is change in leadership at the post graduate institute or if the key analysts switch positions or do not train their replacements. One is hopeful that the widespread success of simulated cases with feedback and benchmarking obviates this problem. The government – through the Ministry of Health – along with professional associations and academia – through the Kyrgyz State Medical Institute of Retraining and Further Training named after S.B.Daniyarov – have taken a more ambitious tack to ensure that the serial measurement and teaching accountability will continue by making participation in this program a requirement for ongoing licensure. Clearly there is a need for outcomes data. Some has been collected in the past, providing valuable direction to the first three target areas of the QuICKK project. Collecting future outcomes data has been discussed but it has not been funded. In an ideal setting, there would be pre-post data but even starting a systematic process now would be helpful with the amount of opportunity there is for further improvement and lower variation. The measures do not have to be exhaustive either: we recommend a focus on a handful of utilization metrics such as admissions, unnecessary testing and blood pressure measured plus a few outcome measures such as birth complications, neonatal deaths and BP control. Once initiated this process can be expanded as resources and clinical urgencies dictate. Notwithstanding there is now a body of work that shows that serial measurement with feedback and benchmarking results in more evidence-based practice [14-18] and better outcomes [23].

In closing, one of the most impressionable findings locally, was the enormous amount of inefficiency observed with low quality high variation care. If the unnecessary testing alone was reduced by 50% this would decrease local health care spending by 63 million som per year. We are among many who feel that improving quality is money well spent but this finding from Kyrgyzstan underscores that more than the foundational argument of providing better care with serial measurement and feedback, it is financially irresponsible not to do so.

## CONCLUSION

QuICCK serially trained more than 2000 physicians across the Kyrgyz Republic with serial simulated patient cases, called CPVs. At baseline, the quality of care varied widely but rapidly improved across three rounds of measurement as measured by the CPV tool, which also specified specific clinical advances and remaining opportunities. The technology and the capability to use the technology was similarly transferred in just a few months. Among the most striking findings was the enormous waste from unnecessary testing that, if addressed by the national and local government and other groups, could add millions of dollars back into national health care spending. The QuICKK project approach may serve as a template to improve health care quality and reduce variation and costs at a national level in developed- and developing-world countries.

## References

[1] Institute of Medicine. Crossing the Quality Chasm: A New Health System for the 21st Century. Washington, DC: The National Academies Press, 2001. 25057539

[2] Peabody J, Shimkhada R, Adeyi O, Wong H, Broughton E, Kruk M. “Quality of Care”. In: Disease Control Priorities (third edition): Volume 9, Disease Control Priorities, edited by D T Jamison, H Gelband, S Horton, P Jha, R Laxminarayan, C N Mock, R Nugent. Washington, DC: World Bank; 2017.

[3] Peabody JW DeMaria L Nguyen SN Smith O Hoth A Luck J Large-scale evaluation of quality of care in 6 countries of Eastern Europe and Central Asia using Clinical Performance and Value Vignettes. Glob Health Sci Pract. 2017;5:412-29. 10.9745/GHSP-D-17-00044 28963174 PMC5620338

[4] Donabedian A The quality of care. How can it be assessed? JAMA. 1988;260:1743-8. 10.1001/jama.1988.03410120089033 3045356

[5] Fritsche G Peabody J Methods to improve quality performance at scale in lower -, and middle-income countries. J Glob Health. 2018;8:021002. 10.7189/jogh.08.021002 30574294 PMC6286673

[6] Peabody JW Florentino J Shimkhada R Solon O Quimbo S Quality variation and its impact on costs and satisfaction: evidence from the QIDS study. Med Care. 2010;48:25-30. 10.1097/MLR.0b013e3181bd47b2 20009777

[7] United Nations Inter-agency Group for Child Mortality Estimation (UN IGME). Levels & Trends in Child Mortality: Report 2019, Estimates developed by the United Nations Inter-agency Group for Child Mortality Estimation. United Nations Children’s Fund, New York: New York; 2019.

[8] World Health Organization. Trends in maternal mortality 2000 to 2017: estimates by WHO, UNICEF, UNFPA, World Bank Group and the United Nations Population Division. Geneva: World Health Organization; 2019.

[9] Ministry of Health of the Kyrgyz Republic. National Committee on Confidential Enquiry into Maternal Deaths. Inception Report on Confidential Enquiry into Maternal Deaths in the Kyrgyz Republic for 2011-2012. Bishkek: National CEMD Committee; 2014.

[10] Say L Chou D Gemmill A Tuncalp O Moller A-B Daniels J Global causes of maternal death: a WHO systematic analysis. Lancet Glob Health. 2014;2:e323-33. 10.1016/S2214-109X(14)70227-X 25103301

[11] World Health Organization. Highlights on health in Kyrgyzstan. Geneva: World Health Organization; 2005.

[12] Peabody JW Luck J Glassman P Dresselhaus TR Lee M Comparison of vignettes, standardized patients, and chart abstraction: a prospective validation study of 3 methods for measuring quality. JAMA. 2000;283:1715-22. 10.1001/jama.283.13.1715 10755498

[13] Peabody JW Luck J Glassman P Jain S Hansen J Spell M Measuring the quality of physician practice by using clinical vignettes: a prospective validation study. Ann Intern Med. 2004;141:771-80. 10.1001/jama.283.13.1715 15545677

[14] Burgon TB Cox-Chapman J Czarnecki C Kropp R Guerriere R Paculdo D Engaging primary care providers to reduce unwanted clinical variation and support ACO cost and quality goals: a unique provider-payer collaboration. Popul Health Manag. 2019;22:321-9. 10.1089/pop.2018.0111 30328782

[15] Bergmann S Tran M Robison K Fanning C Sedani S Ready J Standardising hospitalist practice in sepsis and COPD care. BMJ Qual Saf. 2019;28:800-8. 10.1136/bmjqs-2018-008829 30894422

[16] Weems L Strong J Plummer D Martin J Zweng T Lindsay J A quality collaboration in heart failure and pneumonia inpatient care at Novant Health: standardizing hospitalist practices to improve patient care and system performance. Jt Comm J Qual Patient Saf. 2019;45:199-206. 10.1016/j.jcjq.2018.09.005 30391372

[17] Oravetz P White C Carmouche D Donaldson D Ruhl M Paculdo D Standardising practice in cardiology: reducing clinical variation and cost at Ochsner Health System. Open Heart. 2019;6:e000994. 10.1136/openhrt-2018-000994 30997137 PMC6443124

[18] Yurso M Box B Burgon T Hauck L Tagg K Clem K Reducing unneeded clinical variation in sepsis and heart failure care to improve outcomes and reduce cost: a collaborative engagement with hospitalists in a multistate system. J Hosp Med. 2019;14:E1-6. 10.12788/jhm.3220 31251162 PMC6715052

[19] Peabody JW Liu A A cross-national comparison of the quality of clinical care using vignettes. Health Policy Plan. 2007;22:294-302. 10.1093/heapol/czm020 17660225

[20] Peabody JW Luck J DeMaria L Menon R Quality of care and health status in Ukraine. BMC Health Serv Res. 2014;14:446. 10.1186/1472-6963-14-446 25269470 PMC4263055

[21] Peabody J Shimkhada R Quimbo S Florentino J Bacate MF McCulloch C Financial incentives and measurement improved physicians’ quality of care in the Philippines. Health Aff (Millwood). 2011;30:773-81. 10.1377/hlthaff.2009.0782 21471500

[22] Institute of Medicine (US) Committee on Preventing the Global Epidemic of Cardiovascular Disease. Meeting the Challenges in Developing Countries; Fuster V, Kelly BB, editors. Promoting Cardiovascular Health in the Developing World: A Critical Challenge to Achieve Global Health. Washington (DC): National Academies Press (US); 2010. 20945571

[23] Peabody JW Quimbo S Florentino J Shimkhada R Javier X Paculdo D Comparative effectiveness of two disparate policies on child health: experimental evidence from the Philippines. Health Policy Plan. 2017;32:563-71. 10.1093/heapol/czw179 28110265 PMC5400045

